# An Association between Diet and *MC4R* Genetic Polymorphism, in Relation to Obesity and Metabolic Parameters—A Cross Sectional Population-Based Study

**DOI:** 10.3390/ijms222112044

**Published:** 2021-11-07

**Authors:** Edyta Adamska-Patruno, Witold Bauer, Dorota Bielska, Joanna Fiedorczuk, Monika Moroz, Urszula Krasowska, Przemyslaw Czajkowski, Marta Wielogorska, Katarzyna Maliszewska, Sylwia Puckowska, Lukasz Szczerbinski, Danuta Lipinska, Maria Gorska, Adam Kretowski

**Affiliations:** 1 Clinical Research Centre, Medical University of Bialystok, Marii Sklodowskiej-Curie 24A, 15-276 Bialystok, Poland; witold.bauer@umb.edu.pl (W.B.); urszula.krasowska@umb.edu.pl (U.K.); przemyslaw.czajkowski@umb.edu.pl (P.C.); sylwia.puckowska@umb.edu.pl (S.P.); lukasz.szczerbinski@umb.edu.pl (L.S.); adam.kretowski@wp.pl (A.K.); 2 Department of Family Medicine, Medical University of Bialystok, Mieszka I 4b, 15-054 Bialystok, Poland; d.bielska1@wp.pl; 3 Clinical Research Centre, Medical University of Bialystok Clinical Hospital, Marii Sklodowskiej-Curie 24A, 15-276 Bialystok, Poland; j.fiedorczuk@wp.pl (J.F.); monika_bakun@wp.pl (M.M.); 4 Department of Endocrinology, Diabetology and Internal Medicine, Medical University of Bialystok, Marii Sklodowskiej-Curie 24A, 15-276 Bialystok, Poland; mj.wielogorska@gmail.com (M.W.); maliszewska.k@gmail.com (K.M.); lipinska.danuta11@gmail.com (D.L.); mgorska25@wp.pl (M.G.)

**Keywords:** *MC4R* gene, gene–diet interactions, obesity, obesity-related metabolic complications, macronutrients intake

## Abstract

The melanocortin-4 receptor (MC4R) gene harbours one of the strongest susceptibility loci for obesity and obesity-related metabolic consequences. We analysed whether dietary factors may attenuate the associations between *MC4R* genotypes and obesity and metabolic parameters. In 819 participants genotyped for common *MC4R* polymorphisms (rs17782313, rs12970134, rs633265, and rs135034), the anthropometric measurements, body fat content and distribution (visceral and subcutaneous adipose tissue, VAT and SAT, respectively), and blood glucose, insulin, total-, LDL-, HDL-cholesterol, triglycerides concentrations, and daily macronutrient intake were assessed. ANOVA or Kruskal–Wallis tests were used, and multivariate linear regression models were developed. We observed that the CC genotype carriers (rs17782313) presented higher VAT, VAT/SAT ratio, fasting blood glucose and triglyceride concentrations when they were stratified to the upper quantiles of protein intake. An increase in energy derived from proteins was associated with higher BMI (Est. 5.74, R^2^ = 0.12), body fat content (Est. 8.44, R^2^ = 0.82), VAT (Est. 32.59, R^2^ = 0.06), and VAT/SAT ratio (Est. 0.96, R^2^ = 0.05). The AA genotype carriers (rs12970134) presented higher BMI, body fat, SAT and VAT, fasting blood glucose, triglycerides and total cholesterol concentrations. An increase in energy derived from proteins by AA carriers was associated with higher VAT (Est.19.95, R^2^ = 0.06) and VAT/SAT ratio (Est. 0.64, R^2^ = 0.05). Our findings suggest that associations of the common *MC4R* SNPs with obesity and its metabolic complications may be dependent on the daily dietary intake, which may open new areas for developing personalised diets for preventing and treating obesity and obesity-related comorbidities.

## 1. Introduction

Obesity is a major risk factor for type 2 diabetes mellitus (T2DM) [1], hyperlipidaemia, hypertension, and cardiovascular disease [2]. Obesity has become a major global health challenge [3], and with an increasing prevalence of obesity, increases in the prevalence of these diseases can be expected [4]. Excessive diet energy intake and diminished physical activity contribute to obesity development, but other factors are likely involved [5]. With obesity, many genetic loci and single nucleotide polymorphisms (SNPs) have been associated as well [6,7,8,9,10], mostly due to larger amounts of consumed food [11], deprivations of appetite [12], and substrate utilisation [13,14]. Nevertheless, obesity should not be considered only to be a genetic disorder, because it is a multifactorial disease influenced by the interaction between lifestyle and genetic factors [15]. The melanocortin-4 receptors (MC4R), expressed in several sites in the brain, have been implicated in the central energy balance regulation [16,17]. Activation of the melanocortin system increases energy expenditure and insulin sensitivity, and may influence food intake [8,18]. Rare mutations in the *MC4R* gene have consistently been associated with monogenic obesity [19,20,21]; however, most cases of obesity and T2DM result from polygenic and multifactorial interactions, including *MC4R* gene SNPs [22,23,24,25]. Several studies have demonstrated that SNPs near the *MC4R* gene influence appetite [26], energy and macronutrients intake [23,26]; however, other studies have indicated that SNPs near the *MC4R* gene do not influence food intake [27] and may not have any impact on body weight [28]. These conflicting results may be obtained due to the fact that the effects of *MC4R* may depend on dietary intake, since *MC4R* expression has been found to be associated with the carbohydrates and fat intake [29]. The interactions between *MC4R* genetic variants and dietary factors may play a significant role in the development of obesity and type 2 diabetes phenotypes [25], which highlight the need for additional research on this topic. Therefore, we hypothesise that the association of the *MC4R* gene and obesity and related metabolic parameters may be contingent upon dietary factors, which could explain some of the conflicting results obtained from different studies, and may be a significant direction in obesity prevention and treatment.

## 2. Results

### 2.1. The Characteristic of Studied Population

In this study, 819 subjects (47.5% men and 52.5% women) 18–79 years old were included. The mean age of participants was 42.1 (±14.5) years old. The mean BMI was 28.5 (±6.6) kg/m^2^ (min. 15.6 kg/m^2^, max. 56.5 kg/m^2^). Of the participants, 33.9% had a BMI < 25.00 kg/m^2^, 34.5% were overweight with a BMI ≥ 25.00 and <30.00 kg/m^2^, and 31.6% were obese with BMI ≥ 30.00 kg/m^2^. Clinical characteristic of studied population is presented in Table 1. Based on criteria for diagnosing [30], 411 participants (50.2%) were identified as having prediabetes or diabetes. Of these individuals, 109 participants (13.3%) had a previous history of prediabetes or diabetes, and 56 participants (6.8%) were being treated with anti-diabetic medications. Participants who received anti-diabetic drug therapy or lipid-lowering medications (47 individuals, 5.7% of participants) were excluded from analysis.

The clinical characteristics of the participants, stratified by investigated genotypes, are presented in Table 2, Table 3, Table 4 and Table 5. No significant deviation from the Hardy–Weinberg equilibrium was observed for any of the SNPs investigated in this study (*p* > 0.05). The frequencies of overweight/obesity and prediabetes/diabetes prevalence did not differ between genotypes (Table 2, Table 3, Table 4 and Table 5). We did not find any differences in dietary factors, physical activity level, or body fat distribution between carriers of the investigated genotypes (Table 2, Table 3, Table 4 and Table 5). Additionally, we did not observe significant differences in HbA1c, HOMA-IR, HOMA-B, triglycerides, total cholesterol, LDL cholesterol, or HDL cholesterol levels (data not shown) among individuals carrying investigated SNPs.

### 2.2. Dietary Intake

We did not notice any differences between studied genotypes in daily total energy and macronutrients intake (Table 2, Table 3, Table 4 and Table 5). 

### 2.3. Associations between the rs17782313 Polymorphism and Obesity, Its Comorbidities and Dietary Intake

The CC genotype carriers presented the highest BMI and the highest fasting blood glucose levels (Table 2). Further analysis showed that CC genotype carriers had significantly lower SAT (*p* = 0.029), higher VAT (*p* = 0.03), VAT/SAT ratio (*p* = 0.029), fasting blood glucose (*p* = 0.044), and triglyceride levels (*p* = 0.037), compared with the TT genotype carriers. 

**Figure 1:**
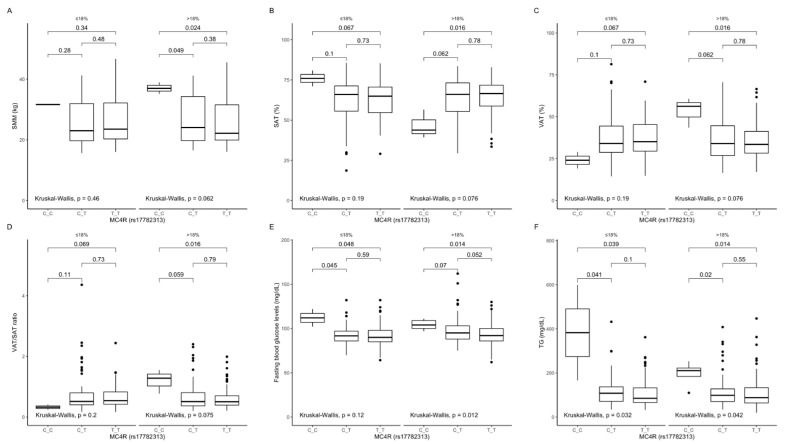
Association of *MC4R* rs17782313 genotypes with (**A**) SMM (kg), (**B**) SAT (%), (**C**) VAT (%), (**D**) VAT/SAT ratio; (**E**) fasting blood glucose levels (mg/dL); (**F**) TG concentrations (mg/dL), by dietary protein strata: ≤18% and >18% of total daily energy intake. SMM, skeletal muscle mass; SAT, subcutaneous adipose tissue; TG, triglycerides; VAT, visceral adipose tissue.

Comparison between carriers of the different genotypes, stratified by macronutrient intake. Among participants in the upper protein intake quantiles, we noted that CC genotype carriers had higher skeletal muscle mass (SMM) content (Figure 1A) and lower SAT (Figure 1B); however, they had higher VAT (Figure 1C), VAT/SAT ratio (Figure 1D), fasting blood glucose (Figure 1E) as well as triglyceride (Figure 1F) levels. We could not analyse participants from the lower protein intake quantiles due to too few genotype CC carriers in this group.

**Figure 2:**
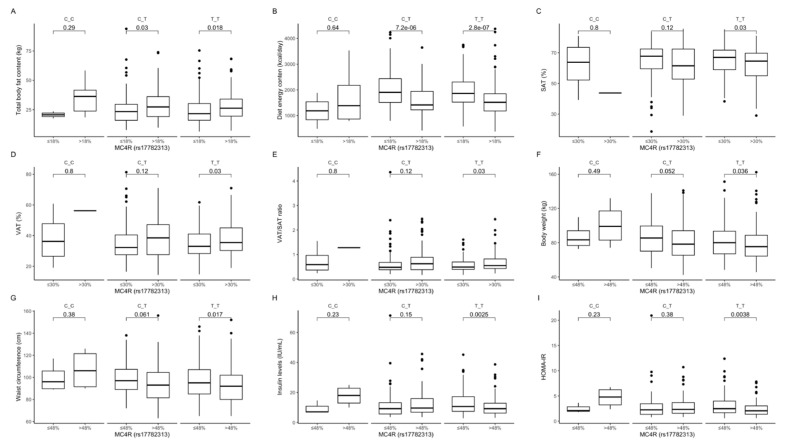
Association of dietary protein intake ≤18% and > 18% of total daily energy intake with (**A**) total body fat content (kg) and (**B**) diet energy content (kcal/day), in *MC4R* rs17782313 genotype carriers. Association of dietary fat intake ≤ 30% and >30% of total daily energy intake with (**C**) SAT (%), (**D**) VAT (%), and (**E**) VAT/SAT ratio, in *MC4R* rs17782313 genotype carriers. Association of dietary carbohydrates intake ≤48% and >48% of total daily energy intake with (**F**) body weight (kg), (**G**) waist circumference (cm), (**H**) fasting insulin levels (IU/mL), and (**I**) HOMA-IR, in *MC4R* rs17782313 genotype carriers. HOMA-IR, Homeostatic Models Assessment of Insulin Resistance; SAT, subcutaneous adipose tissue; VAT, visceral adipose tissue.

Comparison between carriers of the same genotype dependently on macronutrient intake. Comparing participants in the upper protein intake quantiles with participants in the lower protein intake quantiles revealed that higher protein intake was associated with higher total body fat content, also in CT and TT genotype carriers (Figure 2A), and significantly lower diet energy (Figure 2B). Among participants in the upper fat intake quantiles, carrying the TT genotype was associated with lower SAT (Figure 2C) but higher VAT (Figure 2D) and higher VAT/SAT ratio (Figure 2E). Carriers of the TT genotype in the upper carbohydrate intake quantiles had lower body weight (Figure 2F), lower waist circumference (Figure 2G), lower fasting insulin levels (Figure 2H), as well as lower HOMA-IR (Figure 2I) compared to those in the lower carbohydrate intake quantiles. The linear regression models showed that the increase in energy derived from proteins by CC carriers was associated with higher BMIs (Est. 5.74, R^2^ = 0.12, *p* = 0.03), higher body fat content (Est. 8.44, R^2^ = 0.82, *p* = 0.001), higher VAT (Est. 32.59, R^2^ = 0.06, *p* < 0.001), and higher VAT/SAT ratios (Est. 0.96, R^2^ = 0.05, *p* < 0.001). Additionally, in the same group of CC carriers, we observed lower SMM content (Est. −9.97, R^2^ = 0.79, *p* = 0.001) and lower SAT (Est. −32.59, R^2^ = 0.06, *p* < 0.001).

### 2.4. Associations between the rs12970134 Polymorphism and Obesity, Its Comorbidities, and Dietary Intake

We found significant differences in BMI, body fat mass, WHR and fasting blood glucose levels among rs12970134 genotypes (Table 3). Further analysis showed that AA genotype carriers had significantly higher BMIs (AA vs. GG, *p* = 0.0057; AA vs. AG, *p* = 0.046), total body fat content (AA vs. GG, *p* = 0.015; AA vs. AG, *p* = 0.039), SAT (AA vs. GG, *p* = 0.027; AA vs. AG, *p* = 0.029), and waist circumferences (AA vs. GG, *p* = 0.014).

**Figure 3:**
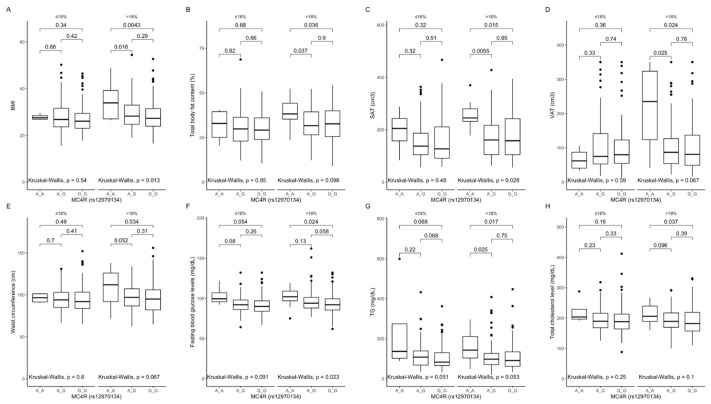
Association of *MC4R* rs12970134 genotypes with (**A**) BMI (kg/m^2^), (**B**) total body fat content (%), (**C**) SAT (cm^3^), (**D**) VAT (cm^3^), (**E**) waist circumference (cm), (**F**) fasting blood glucose levels (mg/dL), (**G**) TG concentrations (mg/dL), and (**H**) total cholesterol concentrations (mg/dL), by dietary protein strata: ≤18% and >18% of total daily energy intake. BMI, Body Mass Index; SAT, subcutaneous adipose tissue; TG, triglycerides; VAT, visceral adipose tissue.

Comparison between carriers of the different genotypes, stratified by macronutrients intake. In participants in the upper protein intake quantiles, we found that AA genotype carriers had higher BMI (Figure 3A), percentage of total body fat content (Figure 3B), SAT (Figure 3C) and VAT volume (Figure 3D), waist circumference (Figure 3E), fasting blood glucose (Figure 3F), triglyceride levels (Figure 3G), and total cholesterol levels (Figure 3H).

**Figure 4:**
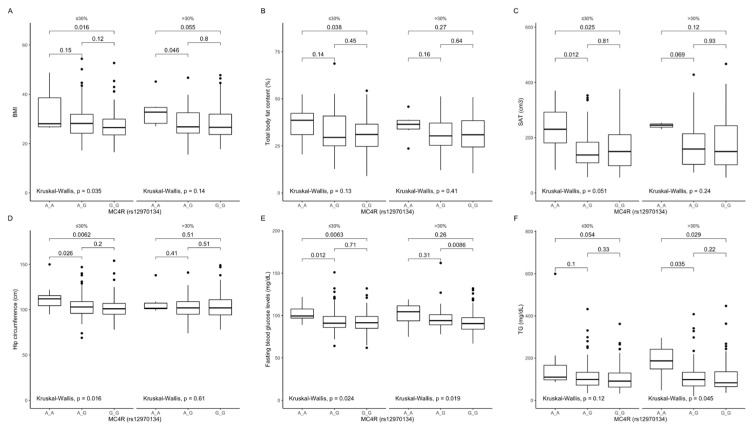
Association of *MC4R* rs12970134 genotypes with (**A**) BMI (kg/m^2^); (**B**) total body fat content (%); (**C**) SAT (cm^3^), (**D**) hip circumference (cm), (**E**) fasting blood glucose concentrations (mg/dL), and (**F**) TG concentrations (mg/dL), by dietary fat intake strata: ≤30% and >30% of total daily energy intake. BMI, Body Mass Index; SAT, subcutaneous adipose tissue; TG, triglycerides.

**Figure 5:**
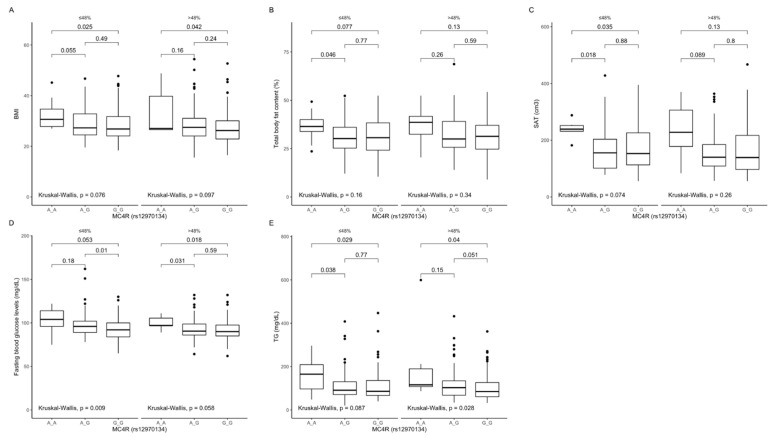
Association of *MC4R* rs12970134 genotypes with (**A**) BMI (kg/m^2^), (**B**) total body fat content (%), (**C**) SAT (cm^3^), (**D**) fasting blood glucose concentrations (mg/dL), and (**E**) TG concentrations (mg/dL), by dietary carbohydrates intake strata: ≤48% and >48% of total daily energy intake. BMI, Body Mass Index; SAT, subcutaneous adipose tissue; TG, tri-glycerides.

We could not analyse participants in the lower protein intake quantiles due to too few AA genotype carriers. We also noted that in AA genotype carriers, these parameters were higher, mostly independent of dietary fat (Figure 4A–F) and dietary carbohydrates (Figure 5A–E). 

**Figure 6:**
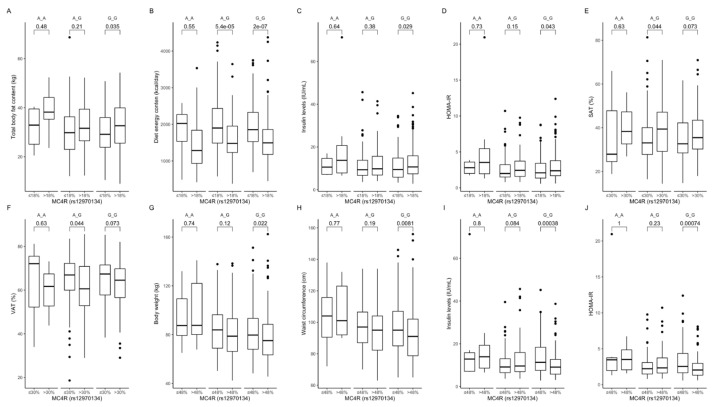
Association of dietary protein intake ≤ 18% and >18% of total daily energy intake with (**A**) total body fat content (kg), (**B**) diet energy content (kcal/day), (**C**) insulin levels (IU/mL), and (**D**) HOMA-IR in *MC4R* rs12970134 genotype carriers. Association of dietary fat intake ≤ 30% and >30% of total daily energy intake with (**E**) SAT (%) and (**F**) VAT (%), in *MC4R* rs12970134 genotype carriers. Association of dietary carbohydrates intake ≤ 48% and >48% of total daily energy intake with (**G**) body weight (kg), (**H**) waist circumference (cm), (**I**) fasting insulin levels (IU/mL), and (**J**) HOMA-IR, in *MC4R* rs12970134 genotype carriers. HOMA-IR, Homeostatic Models Assessment of Insulin Resistance; SAT, subcutaneous adipose tissue; VAT, visceral adipose tissue.

Comparison between carriers of the same genotype dependently on macronutrients intake. In participants being in the higher protein intake quantiles, we noted higher total body fat content in GG genotype carriers (Figure 6A), a lower total energy intake in AG and GG participants (Figure 6B), and higher fasting insulin levels (Figure 6C) and HOMA-IR values (Figure 6D) in GG genotype carriers, when compared with carriers of the same genotype but in lower protein intake quantiles. AG and GG genotype carriers in the upper fat intake quantiles had lower SAT (Figure 6E) and higher VAT (Figure 6F), whereas in the upper carbohydrate intake quantiles they presented lower body weight (Figure 6G), waist circumference (Figure 6H), and fasting insulin levels (Figure 6I), as well as HOMA-IR values (Figure 6J). We could not analyse participants in the lower protein intake quantiles due to too few AA genotype carriers. The linear regression models showed that the increase in energy derived from proteins by AA rs12970134 carriers was associated with lower SMM content (Est. −6.38, R^2^ = 0.79, *p* = 0.016) and SAT (Est. −32.59, R^2^ = 0.06, *p* = 0.03) but higher VAT (Est. 19.95, R^2^ = 0.06, *p* = 0.003) and VAT/SAT ratio (Est. 0.64, R^2^ = 0.05, *p* = 0.006).

### 2.5. Associations between the rs633265 Polymorphism and Obesity, Its Comorbidities and Dietary Intake

We observed that carriers of the GG rs633265 genotype (Table 4) had the highest blood glucose levels at 30 min of OGTT; no other differences were observed among genotypes. Further analysis showed that GG carriers had significantly lower BMI values than TT carriers (*p* = 0.049).

**Figure 7:**
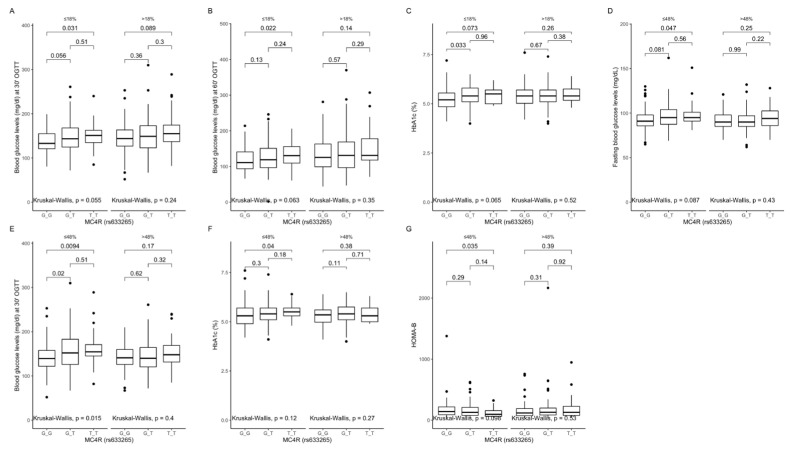
Association of *MC4R* rs633265 genotypes with (**A**) blood glucose levels (mg/dL) at 30 min of OGTT, (**B**) blood glucose levels (mg/dL) at 60 min of OGTT, and (**C**) HbA1c levels (%), by dietary protein intake strata: ≤18% and >18% of total daily energy intake. Association of *MC4R* rs633265 genotypes with (**D**) fasting blood glucose levels (mg/dL), (**E**) blood glucose levels (mg/dL) at 30 min of OGTT, (**F**) HbA1c levels (%), and (**G**) HOMA-B (%), by dietary carbohydrates intake strata: ≤48% and >48% of total daily energy intake. HOMA-B, Homeostatic Models Assessment of β-cell function; OGTT, Oral Glucose Tolerance Test.

Comparison between carriers of the different genotypes, dependently on macronutrient intake. The GG genotype carriers had lower blood glucose levels at 30 min (Figure 7A) and 60 min (Figure 7B) of OGTT, and lower HbA1c (Figure 7C), when they were stratified to the lower protein intake quantiles. Lower blood glucose levels at fasting (Figure 7D) and 30 min of OGTT (Figure 7E), lower HbA1c levels (Figure 7F), and higher HOMA-B values (Figure 7G) were noted also, when they were stratified to the lower carbohydrate intake quantiles.

**Figure 8:**
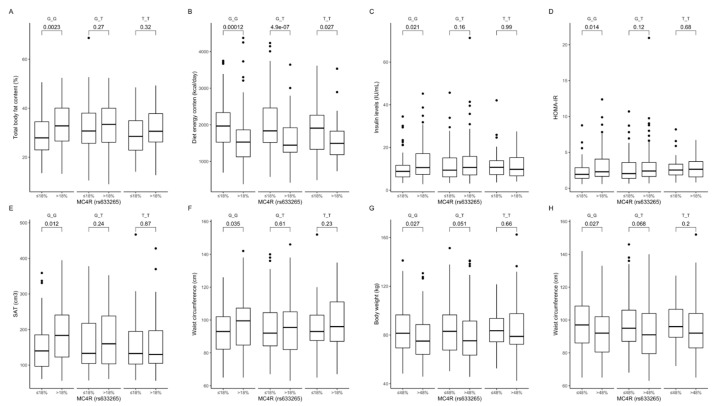
Association of dietary protein intake ≤ 18% and >18% of total daily energy intake with (**A**) total body fat content (%), (**B**) diet energy content (kcal/day), (**C**) fasting insulin levels (IU/mL), (**D**) HOMA-IR, (**E**) SAT (cm^3^), and (**F**) waist circumference (cm), in *MC4R* rs633265 genotype carriers. Association of dietary carbohydrate intake ≤ 48% and >48% of total daily energy intake with (**G**) body weight (kg) and (**H**) waist circumference (cm), in *MC4R* rs633265 genotype carriers. HOMA-IR, Homeostatic Models Assessment of Insulin Resistance; SAT, subcutaneous adipose tissue.

Comparison between carriers of the same genotype dependently on macronutrients intake. The GG genotype carriers in the upper protein intake quantiles had higher percentages of total body fat content (Figure 8A), and lower diet total energy intake (Figure 8B), which was observed in all subjects independent of genotypes. Additionally, the GG carriers in the upper protein intake quantiles had higher fasting insulin levels (Figure 8C), HOMA-IR (Figure 8D), volume of SAT (Figure 8E), and waist circumference (Figure 8F). Furthermore, body weight (Figure 8G) and waist circumference (Figure 8H) were lower when GG genotype carriers were stratified to the upper carbohydrate intake quantiles, even if diets did not differ in total energy content (data not shown).

### 2.6. Association between the rs1350341 Polymorphism and Obesity, Its Comorbidities, and Dietary Intake

The AA rs1350341 carriers had the highest blood glucose levels at 30 min of OGTT (Table 5); no differences were observed among other genotypes. Further analysis showed that GG carriers had significantly lower BMI values than individuals with the AA genotype (*p* = 0.049). 

**Figure 9:**
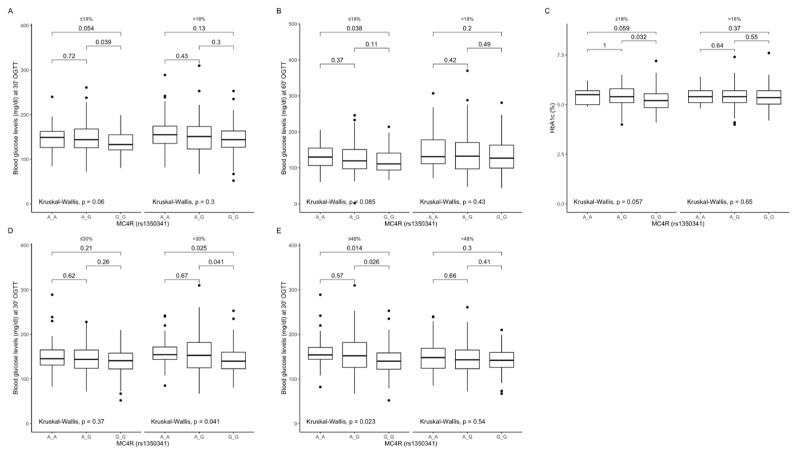
Association of *MC4R* rs1350341 genotypes with (**A**) blood glucose levels (mg/dL) at 30 min of OGTT, (**B**) blood glucose levels (mg/dL) at 60 min of OGTT and (**C**) HbA1c levels (%), by dietary protein intake strata: ≤18% and >18% of total daily energy intake. Association of *MC4R* rs1350341 genotypes with (**D**) blood glucose levels (mg/dL) at 30 min of OGTT, by dietary fat intake strata: ≤30% and >30% of total daily energy intake. Association of *MC4R* rs1350341 genotypes with (**E**) blood glucose levels (mg/dL) at 30 min of OGTT, by dietary carbohydrates intake strata: ≤48% and >48% of total daily energy intake. OGTT, Oral Glucose Tolerance Test.

Comparison between carriers of the different genotypes, stratified by macronutrient intake. Among participants from the lower quantiles of protein intake, we found that GG carriers had lower blood glucose levels at 30 min (Figure 9A) and at 60 min (Figure 9B) of OGTT, as well as lower HbA1c (Figure 9C). Additionally, GG carriers from the upper fat intake quantiles and the lower carbohydrate intake quantiles had significantly lower blood glucose levels at 30 min of OGTT (Figure 9D and Figure 9E, respectively).

**Figure 10:**
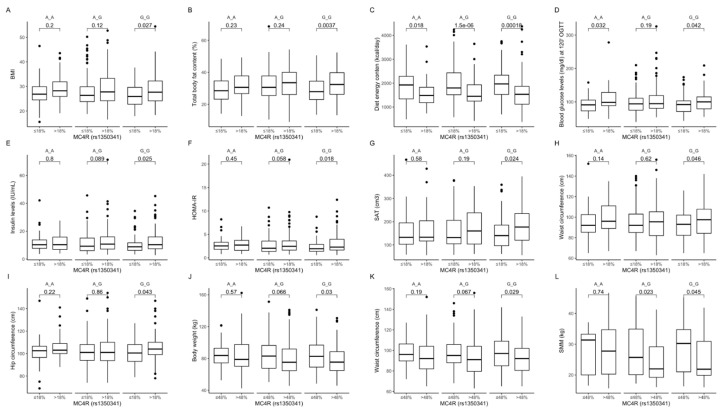
Association of dietary protein intake ≤ 18% and >18% of total daily energy intake with (**A**) BMI (kg/m^2^); (**B**) total body fat content (%); (**C**) diet energy content (kcal/day); (**D**) blood glucose levels (mg/dL) at 120 min of OGTT; (**E**) fasting insulin levels (IU/mL); (**F**) HOMA-IR; (**G**) SAT (cm^3^), (**H**) waist circumference (cm); and (**I**) hip circumference (cm), in *MC4R* rs1350341 genotype carriers. Association of dietary carbohydrates intake ≤ 48% and >48% of total daily energy intake with (**J**) body weight (kg); (**K**) waist circumference (cm); and (**L**) SMM (kg) in *MC4R* rs1350341 genotype carriers. BMI, Body Mass Index; HOMA-IR, Homeostatic Models Assessment of Insulin Resistance; OGTT, Oral Glucose Tolerance Test; SAT, subcutaneous adipose tissue; SMM, skeletal muscle mass.

Comparison between carriers of the same genotype, dependently on macronutrients intake. The GG carriers from the upper quantiles had higher BMI values (Figure 10A) and higher percentage of body fat content (Figure 10B), and lower diet total energy intake (Figure 10C), which was noted for all subjects independently of genotypes. Blood glucose levels at 120 min of OGTT were significantly higher in both GG and AA genotype carriers (Figure 10D) from the upper protein intake quantiles. Moreover, in GG carriers we also noted higher fasting insulin levels (Figure 10E), HOMA-IR (Figure 10F), subcutaneous fat tissue volumes (Figure 10G), as well as waist (Figure 10H) and hip (Figure 10I) circumferences. Body weight (Figure 10J), waist circumference (Figure 10K), and muscle mass (Figure 10L) were lower when GG carriers were stratified to the upper carbohydrate intake quantiles. We also observed lower skeletal muscle mass in AG and GG genotype carriers in the upper carbohydrate intake quantiles (Figure 10L).

## 3. Discussion

The central melanocortin system regulates energy balance by influencing food intake, energy expenditure, and glycemic homeostasis, also independent of its effects on body weight and composition [16]. Our study demonstrated that the effects of *MC4R* genetic variants on body mass, body composition, as well as some metabolic parameters, may depend on dietary factors. In our study, participants did not differ by genotype in daily energy and macronutrient intake and physical activity levels; therefore, we can exclude the impact of these factors on *MC4R* gene expression and activation of melanocortin pathways, as has been previously reported by Lauria et al. [29]. We observed associations between SNPs in rs17782313 with BMI and body fat distribution, fasting blood glucose, and triglycerides levels. Our results are in line with a study by Qi et al. [23], and partially with a study by Zobel et al. [31], who found associations between rs17782313 genotypes and BMI, but did not observe any differences in glucose homeostasis. In our previous study, we observed differences in body fat distribution without finding any differences in BMI between genotypes [32]. These discrepancies might have arisen from differences in the groups chosen for analysis, especially if only women or only men were enrolled in the study, since the body fat content and body fat distribution, as well as its relation to metabolic health in men and women may differ significantly and mostly due to the sex differences in the adipose tissue biology [33]. Nevertheless, these discrepancies prompted us to investigate also other factors that might influence our results. We found metabolic disturbances that are significantly different in individuals from the upper dietary protein intake. It is worth to notice that the mean energy value of diets from upper quantiles of protein intake was significantly lower compared with the lower intake quantiles across all studied genotypes examined. Our results are in contrast with findings from Qi et al. [23], who observed significant differences between rs17782313 genotypes in total energy, fat and protein intake, as well as independent of dietary intake association with BMI. However, only women were included in that study, and there may be both significant sex differences in dietary intakes as well as sexually dimorphic effects of diet on health [34]. In actual fact, we could not exclude the genotype differences also in the participants in the lower protein intake quantiles, since we could not compare it due to too few CC genotype carriers in this group. Moreover, we have noted higher body fat content also among individuals carrying CT and TT genotypes in the upper protein intake quantiles. The linear regression models showed that the increase in the percentage of energy derived from proteins in CC carriers of rs17782313 was associated with higher body mass, body fat, and VAT content. The distribution of body fat tissue is of crucial importance, since visceral adipocytes are more metabolically active, which may lead to the development of insulin resistance (IR), and are associated with all-cause mortality [35,36]. Moreover, higher VAT/SAT ratios may be associated with increased metabolic and cardiovascular risk, independently from BMI and VAT content [37]. Intriguingly, carriers of the protective TT genotype had significantly lower body weight, waist circumference, fasting insulin levels, and HOMA-IR, when in their diet >48% of energy was derived from carbohydrates. This is a very interesting observation, which is salient in light of current interest in carbohydrate-restricted diets among the general population. Surprisingly, our results suggest also that the impact of dietary fat and carbohydrate intake may not be crucial for the risk of obesity in carriers of this high-risk genotype. 

Our study’s associations of the AA genotype of rs12970134 with increased BMI, body fat mass, WHR, and fasting blood glucose levels are in line with our previous findings [32], as well as with results from a study by Zobel et al. [31]. Our study showed that AA genotype carriers following a diet with >18% of total energy from protein had higher VAT, triglycerides, and total cholesterol levels, even if the energy value of the diet was significantly lower. Moreover, our results suggest that participants with the AA genotype had higher values of metabolic parameters, mostly independent from dietary carbohydrate and fat intake. We observed some adverse effect of diet with >18% of energy derived from proteins also in the GG genotype carriers, which suggests that these disadvantageous effects of proteins may be independent from *MC4R* rs12970134 genotypes and may promote insulin resistance also in individuals with the protective genotype. Because we could not analyse these associations between AA genotypes from the lower quantiles of protein intake due to insufficient numbers, we constructed regression models which showed that an increase in the percentage of daily energy delivered from protein in the AA genotype was associated with significantly lower SMM and SAT, but a higher VAT and VAT/SAT ratio. The effects of visceral body fat deposition have been mentioned above, but noted reduction of SMM may also lead to the development of type 2 diabetes [38]. We noticed also that the AG and GG genotype carriers had lower SAT and higher VAT when the percentage of daily energy from fat exceeded 30%. Koochakpoor et al. [25] observed that the risk of abdominal obesity increases across quantiles of total fat intake, only for A allele carriers. However, both, Koochakpoor et al. [25] as well as Murphy AM [37] reported that these kinds of associations may be modulated by saturated fatty acid intake, which was not analysed in our study, and may explain the different results obtained. Wang et al. [26] found that rs12970134 SNPs may possibly increase adiposity by affecting eating behaviours; nevertheless, in our study we observed higher BMI and body fat content without noting any differences in daily energy and macronutrient intake between genotypes.

The other two SNPs in rs633265 and rs1350341 investigated in our study were associated with blood glucose levels during OGTT. We have previously observed that these genetic variants are associated with postprandial glucose utilisation [32]. Comparing genotypes revealed that protective GG genotype of rs633265 carriers had significantly lower BMI, lower blood glucose levels at 30 and at 60 min of OGTT, and lower HbA1c when daily protein intake was lower than 18% of the total energy intake, whereas when dietary proteins provided >18% of daily energy, they presented a higher body fat content, SAT, waist circumference, fasting insulin levels, and HOMA-IR, even if the total energy intake was significantly lower. Moreover, in these participants we observed more favorable results of glucose homeostasis parameters, indicating higher insulin sensitivity and better β-cell function, when the percentage of energy derived from carbohydrates was less than 48%. However, surprisingly, lower body weight and waist circumference were observed when >48% of energy of the GG genotype carriers’ diet was derived from carbohydrates. Similar associations with proteins and carbohydrates intake we noted in homozygous carriers of protective G allele of rs1350341. GG genotype carriers in the upper protein intake quantiles had higher obesity-related parameters, and lower body weight and waist circumference when >48% of total energy was derived from carbohydrates. Considering our previous observations that GG genotype carriers had significantly higher glucose utilisation after a high-carbohydrate meal intake [32]; together with results from this study, we can hypothesise that a mechanism that protects these individuals from de novo lipogenesis and fat deposition may be at play not only after a high-carbohydrate meal intake. Similar to the previously described rs633265, we did not find any other studies that we could compare our results with, because we likely analysed these associations for the first time.

Our study indicates that recommendations to decrease dietary fat < 30% are appropriate for carriers of protective *MC4R* genotypes. It has been found recently also by Rojo D. et al. [39] that the protective mutations of *MC4R* do not protect against diabetogenic and adipogenic effects of a high-fat diet in animal model, which highlights the importance of the proper feeding habits even under favorable genetic conditions. On the other hand, intriguingly, our results suggest that in the carriers of *MC4R* risk genotypes, a dietary total fat intake might not affect metabolic parameters. A previous study by Butler et al. [40] suggested that *MC4R* regulates metabolic and behavioural responses to high-protein and low-fat intake. In our study, carriers of genotypes which predispose individuals to obesity seem to be affected by dietary proteins, and with increasing dietary protein intake the adverse effects may be induced. Additionally, the impact of protective genotypes in rs633265 and rs1350341 may have more beneficial effects on obesity-related comorbidities, especially glucose homeostasis, if dietary protein is maintained at less than 18% of total energy intake. This is a crucial observation from our study, since dietary proteins are considered to be a highly satiating nutrient with some metabolic benefits [41]. 

To the best of our knowledge, this is the first study to investigate interactions between these four common *MC4R* genetic variants and macronutrient intake as well as the effect of these relationships on obesity, body fat content, and obesity-related metabolic consequences. Nevertheless, one must be very cautious when extrapolating our results, since we present analysis for four SNPs of one gene, and there are many other genetic factors (and not only genetic), which may be involved and may play an important role in metabolic pathways. It is necessary to pay attention to the fact that some of these genetic variations are located close to non-coding regions of DNA, and potential associations between adiposity-related traits and non-coding variations are still not clear. Non-coding regions are very intriguing, but mechanisms for these associations have yet to be clarified [42]. It is still not clear if and how the association signals of common non-coding *MC4R* SNPs relate to the receptor gene, but as shown by in vitro assays, the majority of mutations may lead to a partial or total loss of melanocortin 4 receptor function [43,44]. Even though the best models to test gene × environment interactions are randomised clinical trials, the number of participants in such studies are limited, and there is always a risk of false positive findings; therefore, replication in larger and more diverse populations is needed to verify findings. Further studies are needed to determine whether our findings are generalisable to other ethnic groups. A major strength of our study is that it is based on a relatively large sample of both sexes. Nevertheless, it is worth to underline that it has been shown by Vázquez-Moreno M. et al. [45] that sex/gender may also modify the associations between *MC4R* mutations and metabolic disturbances, which highlights the need for further investigations. The dietary data in our study were based on self-reported three-day diaries of food intake, which is a major limitation in our study, because people might tend to underreport their food intake [46]. However, dietary questionnaires and diaries are the only tools that are currently available for large population studies. The other important limitation is that for the daily physical activity evaluation, we could not use accelerometers; nevertheless, the long version of IPAQ questionnaire is a validated method to verify physical activity level, with a high reproducibility.

## 4. Materials and Methods

### 4.1. The Aim and Study Design 

The aim of our study was to evaluate whether dietary factors can alter the impact of the *MC4R* gene on obesity and related metabolic parameters. This study is registered at www.clinicaltrials.gov as NCT03792685-the meal challenge tests study [47,48]; however, data presented in this manuscript are based on a cross-sectional study at baseline, before subjects were included in the intervention sub-study groups.

### 4.2. Participants

A population-based sample was enrolled from the cohort consisting of 1549 Caucasian individuals of Polish origin (aged 18–79 years), recruited as described previously [49,50]. The ancestry of all of the participants was identified by self-identification, and based on the interview. We included subjects without any endocrine, renal, hepatic and gastrointestinal disorders, and treatments (including dietary supplements, following any specific eating patterns, such as vegan, vegetarian, etc.) that might affect the results. Details of the experimental workflow are presented in the Flow Chart (online Supplementary Materials).

### 4.3. Anthropometric Measurements and Body Composition Analysis 

Weight and height were measured using a standardised method [51]. The body composition analysis was performed with the bioelectrical impedance method: total body fat content (InBody 220, Biospace, Seoul, Korea), visceral adipose tissue (VAT) and subcutaneous adipose tissue (SAT) content (Maltron 920-2 BioScan, Maltron International Ltd., Rayleigh, UK). Waist circumference was assessed midway between the lower rib and the iliac crest on the midaxillary line. Hip circumference was measured at the level of the widest circumference over the great trochanters. 

### 4.4. Oral Glucose Tolerance Test (OGTT) Performance 

The OGTTs were performed according to the World Health Organization (WHO) recommendations using a 75g oral glucose dose. All participants were instructed to fast for 8–12 h prior to the tests but not to restrict carbohydrate intake 3 days before the test. The blood was collected 0, 30, 60, and 120 min after glucose load. OGTT was performed in all study participants without a history of diabetes.

### 4.5. Blood Collections and Biochemical Analysis

The specimen was drawn and prepared for testing in accordance with the instructions provided with the laboratory kit. The specimen was stored in accordance with the kit instructions until testing at −20 °C/−80 °C. An immunoradiometric assay (Insulin, IRMA, DiaSource, Belgium; Wallac Wizard 1470 Automatic Gamma Counter, PerkinElmer, Life Science, Turku, Finland) was used to measure insulin concentration. Concentrations of plasma glucose was measured by the hexokinase enzymatic colorimetric assay (Cobas c111, Roche Diagnostics Ltd., Risch-Rotkreuz, Switzerland). Concentrations of fasting triglycerides, total cholesterol, LDL, and HDL were measured with an enzymatic colorimetric assay (Cobas c111, Roche Diagnostics Ltd., Risch-Rotkreuz, Switzerland). HbA1c was assessed using HPLC (high performance liquid chromatography; D-10 Hemoglobin Testing System, Bio-Rad Laboratoriues Inc. Hercules, CA, USA by France, Bio-Rad, Marnes-la-Coquette).

### 4.6. Calculations 

BMI was calculated using the following formula: body weight (kg) divided by height squared (m). Waist–hip ratio (WHR; in centimetres) was determined by dividing waist circumference by hip circumference. VAT/SAT ratio was calculated by dividing visceral adipose tissue by subcutaneous adipose tissue. Homeostatic model assessment of insulin resistance (HOMA-IR) was calculated using the following standard formula: (fasting plasma glucose concentration (mmol/L)) × (fasting insulin concentration (μU/mL))/22.5. The index for homeostatic model assessment of β-cell function (HOMA-B) was determined using the following formula: 20 × fasting insulin (μIU/mL)/fasting glucose (mmol/mL) −3.5. The metabolic equivalent (MET, min per week) was calculated using the following formula: (MET level) × (minutes of activity) × (events per week).

### 4.7. Daily Physical Activity and Dietary Intake Analyses 

Daily physical activity was estimated with a self-administered questionnaire (International Physical Activity Questionnaire-Long Form, IPAQ-LF) a valid metric to assess the levels of physical activity [52]. The results of the questionnaire were used to calculate MET values; each participant was subsequently classified as having a low, moderate, or high physical activity level. Analyses of three-day food diaries were performed in a group of 662 subjects (Supplemetal Figure S1), because not all participants completed the 3-day food intake diary. Participants were asked to compare their portion sizes with colour photographs for each portion size and to weigh their food if possible. Daily energy, protein, fat, and carbohydrate intake were estimated using Dieta 6 software (National Food and Nutrition Institute, Warsaw, Poland).

### 4.8. Study Group Stratification Dependently on the Daily Dietary Intake

In order to study the associations between genetic and dietary factors, the study participants were divided into two quantiles based on the average daily carbohydrates, protein and fat intake as follows: lower and higher than median dietary carbohydrate intake (≤48% and >48% of total energy intake, respectively), lower and higher than median dietary protein intake (≤18% and >18% of total energy intake, respectively), and lower and higher than median dietary fat intake (≤30% and >30% of total energy intake, respectively).

### 4.9. Genetic Analysis 

We genotyped previously identified *MC4R* SNPs in: rs17782313, rs12970134, rs633265, and rs1350341. DNA was extracted from peripheral blood leukocytes using a classical salting out method. The SNPs were genotyped with TaqMan SNP technology from ready-to-use human assays library (Applied Biosystems, Foster City, CA, USA) using a high throughput genotyping system (OpenArray, Life Technologies, Waltham, MA, USA). SNP analysis was performed in duplicate according to the manufacturer’s instructions. To detect possible false positive signals caused by contamination, a negative control consisting of a sample without a template was used. 

### 4.10. Ethics 

Study procedures were carried out in accordance with ethical standards on human experimentation as well as the guidelines laid down in the Helsinki Declaration of 1975, revised in 1983. The trail and study protocol were approved by the local Ethics Committee of the Medical University of Bialystok, Poland (R-I-002/35/2009, 29 January 2009), and written informed consent was obtained from all participants. 

### 4.11. Statistical Analysis 

Numerical data were summarised with the number of observations (*N*), arithmetic mean, and standard deviation (SD). For categorical data, number of observations and percentage were presented. Study participants were divided into quantiles based on their average daily macronutrient intake, using the median values of each three parameters to set the thresholds. The risk genotypes of the investigated common *MC4R* SNPs were predefined based on the literature and our previous findings. Because of the relatively small sample size in our study, we did not include the comparison of the allelic and genotypic frequencies and odds ratio calculations. The chi-squared test was applied to study the differences in the genotype’s frequencies between controls and overweight/obese patients, as well as prediabetic/diabetic subjects (presented in Table 2, Table 3, Table 4 and Table 5). Continuous parameters were tested for normality with a Shapiro–Wilk test, as well as visual inspection. The homogeneity of variance across groups was studied using Levene’s test. Nonparametric tests were used for response variables that failed the mentioned statistical tests. The differences between selected parameters and studied dietary groups were then compared using an analysis of variance (ANOVA) or Kruskal–Wallis test for numerical variables, with Tukey’s or Dunn’s post hoc tests with a Holm *p*-value adjustment where appropriate (in case multiple pairwise tests were performed, or when there were multiple grouping variables, as presented in tables and figures), and with a chi-squared test for categorical variables. To study the hypothesis that the relationship between *MC4R* genotypes and continuous responses varies in average daily protein, carbohydrates, and fat intake groups, we have added (dietary macronutrient quantile) × (genotype) interaction terms to the multivariate linear regression models. These models were adjusted for age, sex, BMI (when applicable), average daily energy intake (kcal/day), and physical activity. The Huber–White robust standard errors (HC1) were calculated. Model fit has been estimated using the R-squared values plus adjusted R-squared values. Some of the models were optimized by a stepwise backward elimination based on the Akaike information criterion (AIC). The statistical significance level was set at <0.05 for all 2-sided tests and multivariate comparisons. All of the calculations were prepared in the R (version 4.0.2) [53].

## 5. Conclusions

We observed that the CC genotype carriers of *MC4R* rs17782313 presented higher visceral adipose tissue content, and a higher VAT/SAT ratio, fasting blood glucose, and triglyceride concentrations when they were stratified to the upper quantiles of protein intake. An increase in energy derived from proteins by these subjects was associated with higher BMI, body fat content VAT, and VAT/SAT ratio. Additionally, the AA genotype carriers of *MC4R* rs12970134 presented higher BMI, total body fat content, subcutaneous and visceral adipose tissue, fasting blood glucose, triglycerides, and total cholesterol concentrations, and an increase in energy derived from proteins by these individuals was associated with higher VAT and VAT/SAT ratio. Our findings suggest that associations of the common *MC4R* SNPs with obesity and its metabolic complications may be dependent on the daily dietary intake, which may explain some of the differences obtained from various studies and may open new areas for developing personalised diets for preventing and treating obesity and obesity-related comorbidities.

## Figures and Tables

**Table 1:** Characteristics of studied population. Data presented as mean and standard deviation (SD), unless otherwise stated. BMI, body mass index, WHR, waist–hip ratio.

Parameter	
*N* (women/men, %)	819 (52.5/47.5)
Age (years)	42.1 (14.5)
BMI (kg/m^2^)	28.5 (6.6)
% of subjects with BMI < 25.0 kg/ m^2^ (%)	33.9
% of subjects with BMI 25.0–29.9 kg/ m^2^ (%)	34.5
% of subjects with BMI ≥ 30.0 kg/ m^2^ (%)	31.6
Fat mass (kg)	27.1 (13.8)
Fat mass (%)	31.4 (9.6)
WHR	0.928 (0.088)
Visceral fat (cm^3^)	108.4 (80.6)
Visceral fat (%)	37.1 (12.1)
Subcutaneous fat (cm^3^)	167.9 (81.7)
Subcutaneous fat (%)	62.8 (12.3)
Visceral/subcutaneous fat ratio	0.669 (0.443)
Frequency of prediabetes or diabetes	
Yes	411 (50.2%)
No	408 (49.8%)
Fasting blood glucose level (mg/dL)	98.8 (23.9)
Daily energy intake (kcal)	1792.5 (697.4)
% of daily energy from protein	18.9 (4.8)
% of daily energy from fat	31.2 (7.5)
% of daily energy from carbohydrates	47.6 (8.6)
Daily physical activity level, *n* (%)	
Low	60 (7.3%)
Moderate	173 (21.1%)
High	586 (71.6%)

**Table 2:** Characteristics of participants stratified by rs17782313 genotypes. Data presented as mean and standard deviation (SD). OGTT, oral glucose tolerance test; WHR, waist–hip ratio.

rs17782313	C/C	C/T	T/T	*p*-Value
*N* (women/men)	30 (10/20)	275 (137/138)	504 (277/227)	
Genotype frequency	3.71%	33.99%	62.30%	>0.05
BMI (kg/m^2^)	29.8 (6.4)	29.0 (6.9)	28.1 (6.5)	*0.049*
BMI < 25.0 (kg/m^2^)	7 (23.3%)	83 (30.5%)	182 (36.4%)	
BMI 25.0–29.9 (kg/m^2^)	9 (30.0%)	98 (36.0%)	170 (34.0%)	0.186
BMI ≥ 30.0 (kg/m^2^)	14 (46.7%)	91 (33.5%)	148 (29.6%)	
Fat mass (kg)	29.2 (12.6)	27.8 (14.6)	26.6 (13.4)	0.322
Fat mass (%)	32.0 (7.7)	31.5 (10.0)	31.3 (9.5)	0.924
WHR	0.944 (0.100)	0.935 (0.087)	0.923 (0.088)	0.133
Visceral fat (cm^3^)	151.9 (114.3)	115.9 (89.7)	101.7 (71.8)	0.264
Visceral fat (%)	42.5 (14.0)	38.1 (13.9)	36.2 (10.7)	0.107
Subcutaneous fat (cm^3^)	179.8 (82.2)	167.7 (79.7)	167.0 (82.8)	0.693
Subcutaneous fat (%)	57.5 (14.0)	61.9 (13.9)	63.6 (11.2)	0.110
Visceral/subcutaneous fat ratio	0.847 (0.478)	0.736 (0.575)	0.623 (0.345)	0.105
Frequency of prediabetes or diabetes				
Yes	19 (61.3%)	148 (53.6%)	241 (47.4%)	0.118
No	12 (38.7%)	128 (46.4%)	267 (52.6%)	
Fasting blood glucose level (mg/dL)	104.5 (31.8)	98.2 (24.0)	94.7 (17.2)	*0.037*
Blood glucose level at 30′ of OGTT (mg/dL)	151.1 (32.0)	148.8 (37.3)	145.2 (35.8)	0.217
Daily energy intake (kcal)	1575.1 (1017.8)	1825.0 (697.5)	1774.6 (689.4)	0.514
% of daily energy from protein	19.8 (2.4)	18.7 (4.3)	19.1 (5.1)	0.329
% of daily energy from fat	27.9 (9.0)	31.1 (6.7)	31.3 (7.9)	0.563
% of daily energy from carbohydrates	50.2 (9.3)	47.6 (8.2)	47.4 (8.9)	0.644
Daily physical activity level				
Low	2 (6.5%)	22 (8.0%)	35 (6.9%)	
Moderate	10 (32.3%)	53 (19.2%)	109 (21.5%)	0.528
High	19 (61.3%)	201 (72.8%)	364 (71.7%)	

**Table 3:** Characteristics of participants stratified by rs12970134 genotypes. Data presented as mean and standard deviation (SD). OGTT, oral glucose tolerance test; WHR, waist–hip ratio.

rs12970134	A/A	A/G	G/G	*p*-Value
*N* (women/men)	44 (18/26)	308 (157/151)	459 (251/208)	
Genotype frequency	5.43%	37.98%	56.59%	>0.05
BMI (kg/m^2^)	30.6 (6.8)	28.8 (6.8)	28.1 (6.5)	*0.010*
BMI < 25.0 (kg/m^2^)	8 (18.2%)	98 (32.1%)	166 (36.5%)	
BMI 25.0–29.9 (kg/m^2^)	15 (34.1%)	105 (34.4%)	157 (34.5%)	0.050
BMI ≥ 30.0 (kg/m^2^)	21 (47.7%)	102 (33.4%)	132 (29.0%)	
Fat mass (kg)	31.3 (13.9)	27.5 (14.1)	26.4 (13.6)	*0.035*
Fat mass (%)	33.7 (8.2)	31.6 (9.8)	31.1 (9.7)	0.209
WHR	0.949 (0.099)	0.936 (0.089)	0.921 (0.086)	*0.021*
Visceral fat (cm^3^)	145.6 (111.7)	111.6 (82.6)	102.9 (75.0)	0.235
Visceral fat (%)	39.4 (15.3)	37.8 (13.1)	36.4 (10.9)	0.685
Subcutaneous fat (cm^3^)	196.4 (74.5)	165.5 (77.2)	167.0 (85.2)	0.075
Subcutaneous fat (%)	60.6 (15.3)	62.2 (13.1)	63.5 (11.4)	0.693
Visceral/subcutaneous fat ratio	0.777 (0.527)	0.712 (0.533)	0.630 (0.355)	0.672
Frequency of prediabetes or diabetes				
Yes	26 (57.8%)	162 (52.3%)	221 (47.8%)	0.285
No	19 (42.2%)	148 (47.7%)	241 (52.2%)	
Fasting blood glucose level (mg/dl)	104.1 (33.1)	97.8 (22.2)	94.5 (17.4)	*0.038*
Blood glucose level at 30′ of OGTT (mg/dl)	148.8 (33.4)	149.3 (36.3)	144.6 (36.4)	0.119
Daily energy intake (kcal)	1599.3 (887.3)	1831.3 (700.8)	1780.0 (683.7)	0.316
% of daily energy from protein	20.5 (4.1)	18.6 (4.1)	19.0 (5.2)	0.130
% of daily energy from fat	29.1 (7.4)	31.1 (7.1)	31.3 (7.7)	0.490
% of daily energy from carbohydrates	47.9 (8.3)	47.9 (8.4)	47.4 (8.8)	0.691
Daily physical activity level				
Low	4 (8.9%)	21 (6.8%)	35 (7.6%)	
Moderate	13 (28.9%)	61 (19.7%)	99 (21.4%)	0.623
High	28 (62.2%)	228 (73.5%)	328 (71.0%)	

**Table 4:** Characteristics of participants stratified by rs633265 genotypes. Data presented as mean and standard deviation (SD). OGTT, oral glucose tolerance test; WHR, waist–hip ratio.

rs633265	G/G	G/T	T/T	*p*-Value
*N* (women/men)	278 (151/127)	399 (213/186)	130 (59/71)	
Genotype frequency	34.45%	49.44%	16.11%	>0.05
BMI (kg/m^2^)	27.9 (6.3)	28.6 (6.8)	28.9 (6.4)	0.134
BMI < 25.0 (kg/m^2^)	100 (36.2%)	140 (35.4%)	32 (24.8%)	
BMI 25.0–29.9 (kg/m^2^)	93 (33.7%)	132 (33.4%)	51 (39.5%)	0.219
BMI ≥ 30.0 (kg/m^2^)	83 (30.1%)	123 (31.1%)	46 (35.7%)	
Fat mass (kg)	26.1 (12.4)	27.5 (14.8)	27.6 (13.1)	0.535
Fat mass (%)	31.1 (9.1)	31.7 (10.3)	31.1 (8.6)	0.786
WHR	0.924 (0.087)	0.928 (0.089)	0.936 (0.088)	0.474
Visceral fat (cm^3^)	101.9 (68.5)	107.0 (82.0)	123.8 (95.4)	0.379
Visceral fat (%)	36.5 (10.9)	36.7 (12.2)	39.4 (13.9)	0.163
Subcutaneous fat (cm^3^)	167.6 (81.4)	166.7 (80.9)	170.5 (84.3)	0.935
Subcutaneous fat (%)	63.5 (10.9)	63.1 (12.8)	60.7 (13.8)	0.169
Visceral/subcutaneous fat ratio	0.635 (0.368)	0.665 (0.484)	0.749 (0.471)	0.164
Frequency of prediabetes or diabetes				
Yes	132 (46.8%)	203 (50.8%)	73 (55.7%)	0.244
No	150 (53.2%)	197 (49.2%)	58 (44.3%)	
Fasting blood glucose level (mg/dL)	98.1 (21.2)	96.8 (22.6)	94.5 (16.7)	0.144
Blood glucose level at 30′ of OGTT (mg/dL)	152.6 (38.7)	147.8 (36.1)	142.0 (34.7)	*0.023*
Daily energy intake (kcal)	1818.8 (740.8)	1796.5 (675.8)	1733.0 (668.6)	0.715
% of daily energy from protein	19.1 (5.3)	18.7 (4.5)	19.2 (4.5)	0.502
% of daily energy from fat	32.1 (8.1)	30.5 (7.1)	31.4 (7.2)	0.120
% of daily energy from carbohydrates	47.0 (9.3)	47.9 (8.4)	47.2 (8.0)	0.535
Daily physical activity level				
Low	15 (5.3%)	36 (9.0%)	9 (6.9%)	
Moderate	66 (23.4%)	79 (19.8%)	26 (19.8%)	0.401
High	201 (71.3%)	285 (71.2%)	96 (73.3%)	

**Table 5:** Characteristics of participants stratified by rs1350341 genotypes. Data presented as mean and standard deviation (SD). OGTT, oral glucose tolerance test; WHR, waist–hip ratio.

rs1350341	A/A	A/G	G/G	*p*-Value
*N* (women/men)	127 (59/68)	390 (207/183)	274 (149/125)	
Genotype frequency	16.06%	49.30%	34.64%	>0.05
BMI (kg/m^2^)	29.0 (6.5)	28.7 (7.0)	27.9 (6.3)	0.128
BMI <25.0 (kg/ m^2^)	31 (24.6%)	136 (35.2%)	100 (36.8%)	
BMI 25.0–29.9 (kg/ m^2^)	51 (40.5%)	131 (33.9%)	91 (33.5%)	0.196
BMI ≥30.0 (kg/ m^2^)	44 (34.9%)	119 (30.8%)	81 (29.8%)	
Fat mass (kg)	27.7 (13.2)	27.4 (15.0)	26.1 (12.5)	0.484
Fat mass (%)	31.2 (8.6)	31.5 (10.3)	31.1 (9.1)	0.890
WHR	0.934 (0.089)	0.927 (0.088)	0.923 (0.087)	0.507
Visceral fat (cm^3^)	124.9 (96.8)	106.1 (81.4)	101.7 (69.1)	0.386
Visceral fat (%)	39.1 (14.0)	36.7 (12.3)	36.6 (11.0)	0.294
Subcutaneous fat (cm^3^)	173.3 (84.9)	165.3 (80.2)	165.8 (80.2)	0.734
Subcutaneous fat (%)	61.0 (13.9)	63.1 (12.9)	63.4 (11.0)	0.304
Visceral/subcutaneous fat ratio	0.741 (0.476)	0.669 (0.490)	0.638 (0.371)	0.296
Frequency of prediabetes or diabetes				
Yes	69 (53.9%)	195 (50.0%)	129 (46.7%)	0.385
No	59 (46.1%)	195 (50.0%)	147 (53.3%)	
Fasting blood glucose level (mg/dl)	97.4 (20.7)	95.9 (17.9)	94.3 (16.2)	0.198
Blood glucose level at 30′ of OGTT (mg/dl)	152.4 (38.7)	148.3 (36.0)	142.0 (34.8)	0.021
Daily energy intake (kcal)	1741.1 (670.7)	1789.2 (674.1)	1823.0 (744.7)	0.742
% of daily energy from protein	19.2 (4.6)	18.7 (4.3)	19.1 (5.4)	0.604
% of daily energy from fat	31.4 (7.1)	30.5 (7.2)	32.1 (8.1)	0.097
% of daily energy from carbohydrates	47.2 (8.1)	48.0 (8.4)	47.0 (9.3)	0.476
Daily physical activity level				
Low	8 (6.2%)	34 (8.7%)	15 (5.4%)	
Moderate	27 (21.1%)	76 (19.5%)	62 (22.5%)	0.524
High	93 (72.7%)	280 (71.8%)	199 (72.1%)	

## Data Availability

This study was retrospectively registered at www.clinicaltrials.gov as NCT03792685, on 3 January 2019.

## Supplementary Materials

The following are available online at https://www.mdpi.com/article/10.3390/ijms222112044/s1.

## Abbreviations

T2DM	Type 2 Diabetes Mellitus
SNPs	single nucleotide polymorphisms
MC4R	melanocortin-4 receptors
VAT	visceral adipose tissue
SAT	subcutaneous adipose tissue
OGTT	Oral glucose tolerance test
WHO	World Health Organization
LDL	low-density lipoprotein
HDL	high-density lipoprotein
HPLC	high performance liquid chromatography
BMI	body mass index
WHR	waist–hip ratio
HOMA-IR	homeostatic model assessment of insulin resistance
HOMA-B	index for homeostatic model assessment of β-cell function
MET	metabolic equivalent
IPAQ-LF	International Physical Activity Questionnaire-Long Form
SD	standard deviation
ANOVA	analysis of variance
SMM	skeletal muscle mass
IR	insulin resistance

## References

[1] Al-Goblan A.S., Al-Alfi M.A., Khan M.Z. (2014). Mechanism linking diabetes mellitus and obesity. Diabetes Metab. Syndr. Obes..

[2] Canto E.D., Ceriello A., Rydén L., Ferrini M., Hansen T.B., Schnell O., Standl E., Beulens J.W. (2019). Diabetes as a cardiovascular risk factor: An overview of global trends of macro and micro vascular complications. Eur. J. Prev. Cardiol..

[3] Jaacks L.M., Vandevijvere S., Pan A., McGowan C., Wallace C., Imamura F., Mozaffarian D., Swinburn B., Ezzati M. (2019). The obesity transition: Stages of the global epidemic. Lancet Diabetes Endocrinol..

[4] Saeedi P., Petersohn I., Salpea P., Malanda B., Karuranga S., Unwin N., Colagiuri S., Guariguata L., Motala A.A., Ogurtsova K. (2019). Global and regional diabetes prevalence estimates for 2019 and projections for 2030 and 2045: Results from the International Diabetes Federation Diabetes Atlas, 9th edition. Diabetes Res. Clin. Pract..

[5] Barness L.A., Opitz J.M., Gilbert-Barness E. (2007). Obesity: Genetic, molecular, and environmental aspects. Am. J. Med Genet. Part A.

[6] Loos R. (2012). Genetic determinants of common obesity and their value in prediction. Best Pr. Res. Clin. Endocrinol. Metab..

[7] Fawcett K.A., Barroso I. (2010). The genetics of obesity: FTO leads the way. Trends Genet..

[8] Adan R.A.H., Tiesjema B., Hillebrand J.J.G., La Fleur S.E., Kas M., De Krom M. (2006). The MC4 receptor and control of appetite. Br. J. Pharmacol..

[9] Mansoori A., Amini M., Kolahdooz F., Seyedrezazadeh E. (2015). Obesity and Pro12Ala Polymorphism of Peroxisome Proliferator-Activated Receptor-Gamma Gene in Healthy Adults: A Systematic Review and Meta-Analysis. Ann. Nutr. Metab..

[10] Rohde K., Keller M., La Cour Poulsen L., Blüher M., Kovacs P., Böttcher Y. (2019). Genetics and epigenetics in obesity. Metabolism.

[11] McCaffery J.M., Papandonatos G.D., Peter I., Huggins G.S., Raynor H., Delahanty L.M., Cheskin L.J., Balasubramanyam A., E Wagenknecht L., Wing R.R. (2012). Obesity susceptibility loci and dietary intake in the Look AHEAD Trial. Am. J. Clin. Nutr..

[12] Wardle J., Carnell S., Haworth C.M., Farooqi I.S., O’Rahilly S., Plomin R. (2008). Obesity associated genetic variation in FTO is associated with diminished satiety. J. Clin. Endocrinol. Metab..

[13] Goran M.I. (1997). Genetic influences on human energy expenditure and substrate utilization. Behav. Genet..

[14] Adamska E., Kretowski A., Goscik J., Citko A., Bauer W., Waszczeniuk M., Maliszewska K., Paczkowska-Abdulsalam M., Niemira M., Szczerbinski L. (2018). The type 2 diabetes susceptibility TCF7L2 gene variants affect postprandial glucose and fat utilization in non-diabetic subjects. Diabetes Metab..

[15] Bienertová-Vasků J., Bienert P., Forejt M., Tomandl J., Brázdová Z., Vasků A. (2010). Genotype x nutrient association of common polymorphisms in obesity-related genes with food preferences and time structure of energy intake. Br. J. Nutr..

[16] Krashes M.J., Lowell B.B., Garfield A.S. (2016). Melanocortin-4 receptor–regulated energy homeostasis. Nat. Neurosci..

[17] Balthasar N., Dalgaard L., Lee C.E., Yu J., Funahashi H., Williams T., Ferreira M., Tang V., McGovern R.A., Kenny C.D. (2005). Divergence of Melanocortin Pathways in the Control of Food Intake and Energy Expenditure. Cell.

[18] Branson R., Potoczna N., Kral J.G., Lentes K.-U., Hoehe M.R., Horber F.F. (2003). Binge Eating as a Major Phenotype of Melanocortin 4 Receptor Gene Mutations. New Engl. J. Med..

[19] Farooqi I.S., O’Rahilly S. (2005). Monogenic Obesity in Humans. Annu. Rev. Med..

[20] Loos R.J.F., Prostate L.T., Lindgren C.M., Li S., Wheeler E., Zhao J.H., Prokopenko I., Inouye M., Freathy R.M., Attwood A.P. (2008). Common variants near MC4R are associated with fat mass, weight and risk of obesity. Nat. Genet..

[21] Ranadive S.A., Vaisse C. (2008). Lessons from Extreme Human Obesity: Monogenic Disorders. Endocrinol. Metab. Clin. N. Am..

[22] Kring I.I.S., Holst C., Toubro S., Astrup A., Hansen T., Pedersen O., Sørensen I.A.T. (2009). Common variants near MC4R in relation to body fat, body fat distribution, metabolic traits and energy expenditure. Int. J. Obes..

[23] Qi L., Kraft P., Hunter D.J., Hu F.B. (2008). The common obesity variant near MC4R gene is associated with higher intakes of total energy and dietary fat, weight change and diabetes risk in women. Hum. Mol. Genet..

[24] Xi B., Takeuchi F., Chandak G.R., Kato N., Pan H.-W., Zhou D.H., Mi J., AGEN-T2D Consortium (2012). Common polymorphism near the MC4R gene is associated with type 2 diabetes: Data from a meta-analysis of 123,373 individuals. Diabetologia.

[25] Koochakpoor G., Hosseini-Esfahani F., Daneshpour M.A., Hosseini S.A., Mirmiran P. (2016). Effect of interactions of polymorphisms in the Melanocortin-4 receptor gene with dietary factors on the risk of obesity and Type 2 diabetes: A systematic review. Diabet. Med..

[26] Wang S., Song J., Yang Y., Chawla N.V., Ma J., Wang H. (2017). Rs12970134 near MC4R is associated with appetite and beverage intake in overweight and obese children: A family-based association study in Chinese population. PLoS ONE.

[27] Hasselbalch A.L., Ängquist L., Christiansen L., Heitmann B.L., Kyvik K.O., Sørensen T.I.A. (2010). A Variant in the Fat Mass and Obesity-Associated Gene (FTO) and Variants near the Melanocortin-4 Receptor Gene (MC4R) Do Not Influence Dietary Intake. J. Nutr..

[28] Muller Y.L., Thearle M.S., Piaggi P., Hanson R., Hoffman D., Gene B., Mahkee D., Huang K., Kobes S., Votruba S. (2014). Common genetic variation in and near the melanocortin 4 receptor gene (MC4R) is associated with body mass index in American Indian adults and children. Qual. Life Res..

[29] Lauria F., Siani A., Pico C., Ahrens W., Bammann K., De Henauw S., Foraita R., Iacoviello L., Kourides Y., Marild S. (2016). A Common Variant and the Transcript Levels of MC4R Gene Are Associated With Adiposity in Children: The IDEFICS Study. J. Clin. Endocrinol. Metab..

[30] American Diabetes Association (2020). Abridged for Primary Care Providers. Clin. Diabetes.

[31] Zobel D.P., Andreasen C.H., Grarup N., Eiberg H., Sørensen T.I., Sandbaek A., Lauritzen T., Borch-Johnsen K., Jørgensen T., Pedersen O. (2008). Variants Near MC4R Are Associated With Obesity and Influence Obesity-Related Quantitative Traits in a Population of Middle-Aged People: Studies of 14,940 Danes. Diabetes.

[32] Adamska-Patruno E., Goscik J., Czajkowski P., Maliszewska K., Ciborowski M., Golonko A., Wawrusiewicz-Kurylonek N., Citko A., Waszczeniuk M., Kretowski A. (2019). The MC4R genetic variants are associated with lower visceral fat accumulation and higher postprandial relative increase in carbohydrate utilization in humans. Eur. J. Nutr..

[33] Karastergiou K., Smith R.S., Greenberg A.S., Fried S.K. (2012). Sex differences in human adipose tissues–The biology of pear shape. Biol. Sex Differ..

[34] Bennett E., Peters A.E.S., Woodward M. (2018). Sex differences in macronutrient intake and adherence to dietary recommendations: Findings from the UK Biobank. BMJ Open.

[35] Ibrahim M.M. (2010). Subcutaneous and visceral adipose tissue: Structural and functional differences. Obes. Rev..

[36] Katzmarzyk P.T., Mire E., Bouchard C. (2012). Abdominal obesity and mortality: The Pennington Center Longitudinal Study. Nutr. Diabetes.

[37] Kaess B.M., Pedley A., Massaro J., Murabito J., Hoffmann U., Fox C.S. (2012). The ratio of visceral to subcutaneous fat, a metric of body fat distribution, is a unique correlate of cardiometabolic risk. Diabetologia.

[38] Maliszewska K., Adamska-Patruno E., Kretowski A. (2019). The interplay between muscle mass decline, obesity, and type 2 diabetes. Pol. Arch. Intern. Med..

[39] Rojo D., McCarthy C., Raingo J., Rubinstein M. (2020). Mouse models for V103I and I251L gain of function variants of the human MC4R display decreased adiposity but are not protected against a hypercaloric diet. Mol. Metab..

[40] Butler A., Marks D.L., Fan W., Kuhn C.M., Bartolome M.V., Cone R.D. (2001). Melanocortin-4 receptor is required for acute homeostatic responses to increased dietary fat. Nat. Neurosci..

[41] Weigle D.S., Breen P.A., Matthys C.C., Callahan H.S., Meeuws K.E., Burden V.R., Purnell J.Q. (2005). A high-protein diet induces sustained reductions in appetite, ad libitum caloric intake, and body weight despite compensatory changes in diurnal plasma leptin and ghrelin concentrations. Am. J. Clin. Nutr..

[42] Meisel S.F., Carere D.A., Wardle J., Kalia S.S., Moreno T.A., Mountain J.L., Roberts J.S., Green R.C., for the PGen Study Group (2015). Explaining, not just predicting, drives interest in personal genomics. Genome Med..

[43] Scherag A., Jarick I., Grothe J., Biebermann H., Scherag S., Volckmar A.-L., Vogel C.I.G., Greene B., Hebebrand J., Hinney A. (2010). Investigation of a Genome Wide Association Signal for Obesity: Synthetic Association and Haplotype Analyses at the Melanocortin 4 Receptor Gene Locus. PLoS ONE.

[44] Hinney A., Bettecken T., Tarnow P., Brumm H., Reichwald K., Lichtner P., Scherag A., Nguyen T.T., Schlumberger P., Rief W. (2006). Prevalence, Spectrum, and Functional Characterization of Melanocortin-4 Receptor Gene Mutations in a Representative Population-Based Sample and Obese Adults from Germany. J. Clin. Endocrinol. Metab..

[45] Vázquez-Moreno M., Locia-Morales D., Valladares-Salgado A., Sharma T., Wacher-Rodarte N., Cruz M., Meyre D. (2021). Sex/Gender Modifies the Association Between the MC4R p.Ile269Asn Mutation and Type 2 Diabetes in the Mexican Population. J. Clin. Endocrinol. Metab..

[46] Heitmann B.L., Lissner L. (1995). Dietary underreporting by obese individuals–is it specific or non-specific?. BMJ.

[47] Adamska-Patruno E., Ostrowska L., Golonko A., Pietraszewska B., Goscik J., Kretowski A., Gorska M. (2018). Evaluation of Energy Expenditure and Oxidation of Energy Substrates in Adult Males after Intake of Meals with Varying Fat and Carbohydrate Content. Nutrients.

[48] Adamska-Patruno E., Ostrowska L., Goscik J., Pietraszewska B., Kretowski A., Gorska M. (2018). The relationship between the leptin/ghrelin ratio and meals with various macronutrient contents in men with different nutritional status: A randomized crossover study. Nutr. J..

[49] Czajkowski P., Adamska-Patruno E., Bauer W., Fiedorczuk J., Krasowska U., Moroz M., Gorska M., Kretowski A. (2020). The Impact of *FTO* Genetic Variants on Obesity and Its Metabolic Consequences Is Dependent on Daily Macronutrient Intake. Nutrients.

[50] Bauer W., Adamska-Patruno E., Krasowska U., Moroz M., Fiedorczuk J., Czajkowski P., Bielska D., Gorska M., Kretowski A. (2021). Dietary Macronutrient Intake May Influence the Effects of TCF7L2 rs7901695 Genetic Variants on Glucose Homeostasis and Obesity-Related Parameters: A Cross-Sectional Population-Based Study. Nutrients.

[51] Nagy E., Vicente-Rodriguez G., Manios Y., Béghin L., Iliescu C., Censi L., Dietrich S., Ortega F.B., De Vriendt T., on behalf of the HELENA Study Group (2008). Harmonization process and reliability assessment of anthropometric measurements in a multicenter study in adolescents. Int. J. Obes..

[52] Hagströmer M., Oja P., Sjöström M. (2006). The International Physical Activity Questionnaire (IPAQ): A study of concurrent and construct validity. Public Health Nutr..

[53] Team R.C. (2020). R: A Language and Environment for Statistical Computing.

