# Supplementary figures and images for: An Association between Diet and MC4R Genetic Polymorphism, in Relation to Obesity and Metabolic Parameters—A Cross Sectional Population-Based Study
Source: Int J Mol Sci. 2021 Nov 7;22(21):12044. doi: 10.3390/ijms222112044 (PMC8584592; doi:10.3390/ijms222112044)

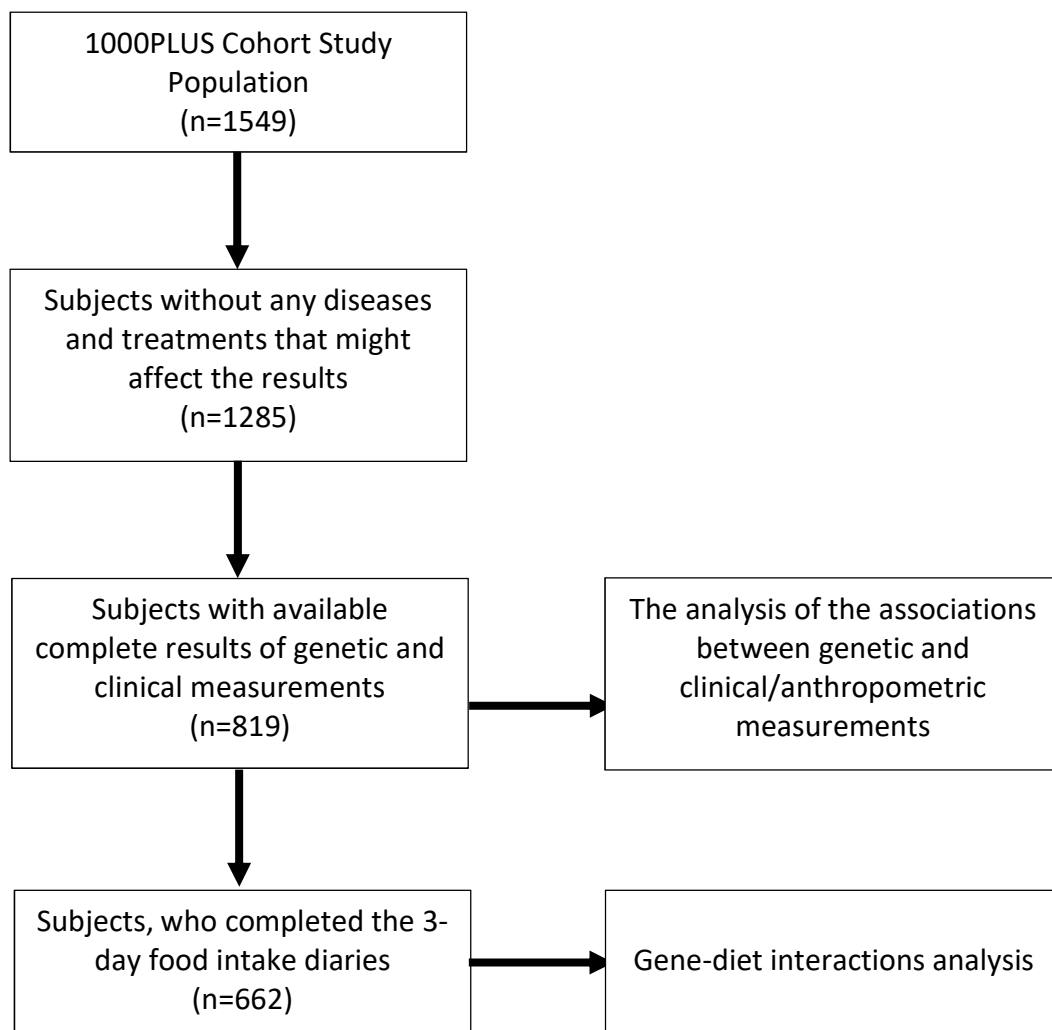

Figure S1. The flow-chart diagram.

Supplement: Supplementary file 1 [file ijms-22-12044-s001.zip › ijms-1433866-supplementary.pdf]
